# Kidney outcomes with GLP-1 receptor agonists in people with type 2 diabetes already receiving SGLT2 inhibitors: a target trial emulation study using UK primary care data

**DOI:** 10.1016/j.lanprc.2026.100139

**Published:** 2026-04

**Authors:** Thijs T Jansz, Andrew P McGovern, Katherine G Young, Martha M Dinsdale, Pedro Cardoso, Beverley M Shields, Andrew T Hattersley, Angus G Jones, Ewan R Pearson, Coralie Bingham, Richard A Oram, John M Dennis

**Affiliations:** a University of Exeter Medical School, University of Exeter, Exeter, UK; b Division of Diabetes, Endocrinology, and Reproductive Biology, Ninewells Hospital and Medical School, University of Dundee, Dundee, UK

## Abstract

**Background:**

Type 2 diabetes is the leading cause of kidney failure and is predominantly managed in primary care. Large randomised trials have shown that GLP-1 receptor agonists and SGLT2 inhibitors each slow kidney disease progression, but their combined effect on kidney outcomes remains unclear. We aimed to evaluate the comparative effectiveness of GLP-1 receptor agonists versus DPP-4 inhibitors or sulfonylureas on kidney outcomes in individuals with type 2 diabetes already receiving SGLT2 inhibitor treatment.

**Methods:**

In this observational study, we emulated a pragmatic target trial using an active-comparator, new-user cohort design with UK primary care electronic health record data (Clinical Practice Research Datalink, March 31, 2013–March 31, 2023) with linkage to hospital inpatient, deprivation, and mortality data. We included individuals with type 2 diabetes already receiving SGLT2 inhibitors who newly initiated either GLP-1 receptor agonists or comparator drugs DPP-4 inhibitors or sulfonylureas. We excluded those with estimated glomerular filtration rate (eGFR) of less than 20 mL/min per 1·73 m^2^ or end-stage kidney disease. Individuals in the comparator group who initiated a GLP-1 receptor agonist during follow-up were censored at the date of GLP-1 receptor agonist initiation and subsequently re-entered into the GLP-1 receptor agonist group. We followed up all initiations regardless of subsequent treatment discontinuation (intention-to-treat analysis), with a maximum follow-up of 3 years. The primary outcome was kidney disease progression (defined as occurrence of ≥40% eGFR decline, end-stage kidney disease, or death from kidney-related causes). Safety outcomes were acute pancreatitis and incident retinopathy (among those without a recorded history at baseline). We estimated hazard ratios (HRs) using Cox proportional hazards models with double-robust overlap weighting.

**Findings:**

We included 33 659 treatment initiations (20 039 GLP-1 receptor agonists and 13 620 DPP-4 inhibitors or sulfonylureas; among 31 650 unique individuals), of whom these initiations occurred among 20 239 (60%) male individuals and 13 420 (40%) female individuals; median age was 60 years (IQR 53–67), and 26 530 (79%) were White, 4492 (13%) south Asian, 1398 (4%) Black, and 1239 (4%) of other ethnicities. Over a median follow-up of 1·4 years (IQR 0·6–3·0), kidney disease progression occurred in 187 (0·9%) of 20 039 individuals who initiated a GLP-1 receptor agonist and 189 (1·4%) of 13 620 who initiated a DPP-4 inhibitor or sulfonylurea. GLP-1 receptor agonist initiation was associated with a lower risk of kidney disease progression compared with DPP-4 inhibitor or sulfonylurea initiation (HR 0·73 [95% CI 0·58–0·92]). GLP-1 receptor agonist initiation was not associated with occurrence of acute pancreatitis (32 [0·2%] of 19 727 individuals with no recorded history of pancreatitis who initiated a GLP-1 receptor agonist *vs* 25 [0·2%] of 13 305 who initiated a DPP-4 inhibitor or sulfonylurea; HR 0·94 [0·52–1·70]) or incident diabetic retinopathy (1097 [10·1%] of 10 844 individuals with no recorded history of diabetic retinopathy who initiated a GLP-1 receptor agonist *vs* 936 [10·7%] of 8719 who initiated a DPP-4 inhibitor or sulfonylurea; HR 1·07 [0·97–1·18]).

**Interpretation:**

These real-world data suggest that the kidney-protective benefits of GLP-1 receptor agonists observed in randomised trials of individuals with type 2 diabetes might also extend to individuals already receiving SGLT2 inhibitors. This finding supports consideration of combination treatment for kidney protection in primary care, particularly in individuals at highest absolute risk. Further prospective data would be valuable to confirm these findings.

**Funding:**

UK Medical Research Council.

## Introduction

Diabetes is the leading cause of chronic kidney disease and kidney failure.^1^ As type 2 diabetes management is predominantly coordinated in primary care, primary care clinicians play a key role in kidney protection. Meta-analyses of large randomised controlled trials have shown that two commonly used glucose-lowering drug classes, SGLT2 inhibitors and GLP-1 receptor agonists, each slow kidney disease progression and reduce cardiovascular events in people with type 2 diabetes,^2^^,^^3^ with SGLT2 inhibitors generally providing stronger kidney protection and consequently being recommended as foundational kidney-protective treatment by guidelines.^4^ However, the combined effect of these drug classes on kidney outcomes is still uncertain.

**Research in context**

**Evidence before this study**

We searched PubMed for articles published from database inception to Oct 1, 2025, using terms “SGLT2” and “GLP1” in combination with “kidney” or “renal”, without language restrictions. We included original research reporting relative risks of adverse kidney outcomes among individuals with type 2 diabetes treated with both GLP-1 receptor agonists and SGLT2 inhibitors, compared with SGLT2 inhibitors alone. One randomised trial (FLOW) comparing GLP-1 receptor agonists versus placebo reported no evidence of effect modification by baseline SGLT2 inhibitor use (p=0·11), although only 16% of participants were on SGLT2 inhibitors, restricting its statistical power. Observational evidence from real-world data has been sparse and heterogeneous. Several cohort studies have suggested a potential benefit of adding GLP-1 receptor agonists to SGLT2 inhibitor treatment, but these analyses were hampered by incomplete adjustment for kidney disease severity (eg, omission of urine albumin–creatinine ratio), comparator groups susceptible to indication bias, and non-standard or poorly defined kidney outcomes. No randomised trials are ongoing or planned to address this question (according to a search of ClinicalTrials.gov on Oct 1, 2025).

**Added value of this study**

This study provides large-scale, real-world evidence that GLP-1 receptor agonist initiation is associated with a lower risk of kidney disease progression than initiation of a DPP-4 inhibitor or sulfonylurea in individuals with type 2 diabetes already receiving SGLT2 inhibitor treatment. These findings suggest that the kidney-protective benefit of GLP-1 receptor agonists could be additive to SGLT2 inhibitor treatment in clinical practice. Furthermore, this lower risk remained consistent across the spectrum of baseline kidney disease severity.

**Implications of all the available evidence**

Our findings are consistent with the kidney-protective benefits of GLP-1 receptor agonists observed in randomised trials, suggesting that these benefits extend to individuals with type 2 diabetes already receiving SGLT2 inhibitor treatment. Current guidelines primarily emphasise adding GLP-1 receptor agonists to SGLT2 inhibitor treatment for cardiovascular protection; however, our findings highlight a potential additional role for kidney protection, particularly in those at highest risk of kidney failure. Future research should focus on the comparative effectiveness of individual GLP-1 receptor agonist preparations for kidney protection, as well as optimal treatment sequencing and combination strategies in prospective studies.

Evidence on the kidney-protective benefits of combined SGLT2 inhibitor and GLP-1 receptor agonist treatment is sparse. The FLOW trial showed that subcutaneous semaglutide reduced the risk of kidney disease progression^5^ but was underpowered to assess whether this effect differed by baseline SGLT2 inhibitor use.^6^ Observational studies suggest additive cardiovascular benefits of combined SGLT2 inhibitor and GLP-1 receptor agonist treatment,^7^, ^8^, ^9^ but evidence for kidney outcomes is sparse. As noted in a recent systematic review, several studies evaluating combination treatment have suggested a possible added benefit of GLP-1 receptor agonists.^10^ However, these studies used non-standard or poorly defined kidney endpoints,^8^^,^^9^^,^^11^ restricting clinical interpretability. One study in people with metabolic dysfunction-associated steatotic liver disease reported a lower risk of major adverse kidney events with combined SGLT2 inhibitor and GLP-1 receptor agonist treatment compared with SGLT2 inhibitors alone,^12^ but was hampered by indication bias because many users of SGLT2 inhibitor did not have type 2 diabetes. Furthermore, all previous studies were restricted by incomplete adjustment for kidney disease severity, eg, urine albumin–creatinine ratio (uACR).

We aimed to address this evidence gap by evaluating the kidney-protective benefit of adding GLP-1 receptor agonists to SGLT2 inhibitor treatment in routine primary care. To do so, we emulated a pragmatic target trial, a framework designed to minimise bias in observational data, comparing initiation of GLP-1 receptor agonists versus neutral comparator glucose-lowering drugs DPP-4 inhibitors and sulfonylureas among individuals with type 2 diabetes already receiving SGLT2 inhibitor treatment.

## Methods

### Study design, population, and data sources

In this observational study, we emulated a pragmatic target trial^13^ using a new-user, active comparator design, comparing initiation of GLP-1 receptor agonists versus DPP-4 inhibitors or sulfonylureas in individuals with type 2 diabetes already receiving SGLT2 inhibitor treatment. We operationalised the target trial specification (appendix pp 2–5) using an active-comparator, new-user design using electronic health record data from the UK-representative Clinical Practice Research Datalink (CPRD) Aurum dataset.^14^ These data were provided prelinked to UK Office for National Statistics cause of death data and, for most participants, to national hospital inpatient (Hospital Episode Statistics) and socioeconomic deprivation (Index of Multiple Deprivation 2019 [IMD]) data.

Using established methods for data extraction and handling,^15^ we included individuals: with type 2 diabetes without previous GLP-1 receptor agonist prescriptions; who were already receiving SGLT2 inhibitor treatment; who received a first-observed prescription for GLP-1 receptor agonists or, as comparator drugs, DPP-4 inhibitors or sulfonylureas, between March 31, 2013, and March 31, 2023; and for whom at least 91 days of registration data were available before first prescription. In the UK, primary care records are the central repository for chronic prescriptions and can be used for identifying treatment initiations in type 2 diabetes.^15^ We excluded individuals meeting any of the following criteria: missing baseline estimated glomerular filtration rate (eGFR) or uACR; eGFR of less than 20 mL/min per 1·73 m^2^ or end-stage kidney disease (requirement for renal replacement therapy or sustained eGFR <15 mL/min per 1·73 m^2^), as SGLT2 inhibitors are not licensed in such cases; or concurrent prescription of dual glucose-dependent insulinotropic polypeptide and GLP-1 receptor agonists.

We defined treatment initiation and study baseline as the date of a first-observed prescription for either a GLP-1 receptor agonist or a comparator drug (DPP-4 inhibitor or sulfonylurea). DPP-4 inhibitors and sulfonylureas were considered a single comparator group because neither alter kidney disease progression,^16^^,^^17^ and to increase statistical power for heterogeneity analyses. All individuals were followed up from drug initiation (baseline) until the earliest of the following: outcome event, death, primary care practice deregistration, end of the study period (March 31, 2023), or a maximum of 3 years (extended to 5 years in sensitivity analyses), using a pragmatic intention-to-treat approach with follow-up continued regardless of whether repeat prescriptions were issued. Individuals in the comparator group who later initiated GLP-1 receptor agonist treatment during follow-up were censored at the date of GLP-1 receptor agonist initiation. These individuals subsequently re-entered the cohort in the GLP-1 receptor agonist group from that date, contributing separate non-overlapping observation periods; the follow-up period was reset at the point of re-entry. Individuals in either group who initiated DPP-4 inhibitors or sulfonylureas treatment after baseline were not censored.

Approval for the study was granted by the CPRD Independent Scientific Advisory Committee (eRAP 24_004747). The CPRD obtains annual research ethics approval from the UK Health Research Authority Research Ethics Committee (05/MRE04/87) to receive and supply patient data for public health research; no further ethical permissions were required for the analyses of these anonymised patient-level data.

The diabetes research cohort within CPRD was developed in consultation with the Exeter Diabetes Patient and Public Involvement Group, who were key in identifying the need for more tailored evidence for the choice of type 2 diabetes treatments. There was no direct patient involvement in the specific design, analysis, or interpretation of this study.

### Exposures and variables

We extracted routinely collected health data from CPRD, including demographic characteristics, diagnoses, prescriptions, laboratory tests, and physiological measurements. All clinical information was identified using standardised clinical codes (Read and SNOMED-CT in primary care, and ICD-10 and OPCS-4 for hospital inpatient data). All codelists are publicly available online.

The primary outcome was kidney disease progression, defined as occurrence of a sustained decline of 40% or more in eGFR (using measurements ≥28 days apart or the final measurement before end of follow-up), end-stage kidney disease (requirement for renal replacement therapy or sustained eGFR <15 mL/min per 1·73 m^2^), or death from kidney-related causes; this definition is consistent with clinical trial and regulatory definitions.^18^ Secondary outcomes were major adverse cardiovascular events (occurrence of myocardial infarction, stroke, or death from cardiovascular causes) and hospitalisation for heart failure. Safety outcomes were acute pancreatitis (in those without previous acute or chronic pancreatitis) and incident diabetic retinopathy (in those without previous diabetic retinopathy). As a neutral control outcome, we evaluated lower limb fracture, because GLP-1 receptor agonists are not known to influence fracture risk.^19^^,^^20^

We predefined a comprehensive set of baseline demographic, clinical, and treatment-related covariates on the basis of clinical knowledge and previous randomised trials. The full covariate set comprised age (years), sex (self-reported: male or female), ethnicity (self-reported and categorised into major UK groups: White, south Asian, Black, or other), IMD quintile (1 being least deprived and 5 most deprived), BMI (kg/m^2^), systolic blood pressure (mm Hg), total cholesterol (mmol/L), glycated haemoglobin (mmol/mol and %), eGFR (calculated using the Chronic Kidney Disease Epidemiology Collaboration formula;^21^ mL/min per 1·73 m^2^), uACR (mg/mmol), duration of type 2 diabetes (years), smoking status (non-smoker, ex-smoker, or current smoker), previous atrial fibrillation (yes or no), atherosclerotic cardiovascular disease (ischaemic heart disease or angina, peripheral vascular disease, revascularisation, stroke, or transient ischaemic attack; yes or no), heart failure (yes or no), Electronic Frailty Index (continuous),^22^ hospitalisation in the year before baseline (yes or no), calendar year at baseline (2013–16, 2017–18, 2019–20, 2021–22, and 2023), number of current glucose-lowering treatments including insulin (2, 3, and ≥4), and concurrent prescriptions (for statins, insulin, and angiotensin-converting enzyme inhibitors or angiotensin-II receptor blockers). For physiological and laboratory measurements, we used the value taken at the date closest to baseline in the previous 2 years.

### Statistical analysis

Our primary causal estimand was the intention-to-treat effect of GLP-1 receptor agonist initiation versus DPP-4 inhibitor or sulfonylurea initiation on the 3-year hazard of kidney disease progression among individuals already receiving SGLT2 inhibitor treatment eligible at time zero. This approach assumed conditional exchangeability, positivity, and correct model specification. We did not perform an a priori sample size calculation.

To mirror the covariate balance attained by randomisation while minimising instability from extreme weights, we used overlap weighting, a propensity score approach that upweights individuals in the region of covariate overlap between treatment groups.^23^ We estimated propensity scores using multivariable logistic regression including the full covariate set (as defined earlier) to meet the conditional exchangeability assumption. We assessed covariate balance using standardised mean differences, with values less than 0·1 indicating satisfactory balance, and confirmed that the positivity assumption was met on the basis of overlap of propensity score distributions (appendix p 20). For all outcomes, we then fitted weighted Cox proportional hazards models with robust variance estimators clustered by patient to estimate hazard ratios (HRs) and 95% CIs. All weighted estimates were double robust to mitigate potential misspecification of either the propensity score or outcome model,^13^ combining weighting with multivariable adjustment using the full covariate set. HRs presented in the text are double-robust overlap weighted unless otherwise specified. We confirmed that proportional hazards assumptions were met on the basis of Schoenfeld residuals. As a post-hoc analysis, we estimated 3-year absolute risk differences using double-robust G computation standardised to the overlap-weighted population.

We tested for effect modification by fitting interaction terms in subgroups defined by sex (male *vs* female), ethnicity (White *vs* not White), eGFR (<60 mL/min per 1·73 m^2^ *vs* ≥60 mL/min per 1·73 m^2^), uACR (<3 mg/mmol *vs* ≥3 mg/mmol), previous atherosclerotic cardiovascular disease (yes *vs* no), and heart failure (yes *vs* no). We also explored non-linear interaction terms for the following continuous covariates using restricted cubic splines with three knots: age at baseline (years), eGFR (mL/min per 1·73 m^2^), uACR (mg/mmol), and predicted 3-year risk of 40% or more eGFR decline or end-stage kidney disease using the Chronic Kidney Disease Prognosis Consortium (CKD-PC) risk score.^24^

We performed the following sensitivity analyses of the primary outcome: we separately evaluated initiation of dulaglutide or subcutaneous semaglutide (which have direct kidney outcome evidence from trials)^5^^,^^25^ and other GLP-1 receptor agonist preparations compared with DPP-4 inhibitors or sulfonylureas initiation; we repeated the primary analysis with follow-up extended to a maximum of 5 years; we did a per-protocol analysis by censoring individuals after treatment discontinuation, defined as no repeat prescriptions after a 183-day grace period; instead of overlap weighting, we used multivariable adjustment only, using the full covariate set; instead of overlap weighting, we used inverse probability of treatment weighting, with weights estimated as described previously and truncated at the 2nd and 98th percentiles; and we did Fine–Gray competing risk regression accounting for death using overlap weighting, providing a subdistribution HR reflecting overall prognostic impact rather than the direct treatment effect. Additionally, we did sensitivity analyses including and excluding specific patient subgroups, as follows: we repeated the primary analysis including estimation of overlap weights after excluding GLP-1 receptor agonist initiations following a previous DPP-4 inhibitor or sulfonylurea initiation during the study period; after excluding individuals treated with insulin; after excluding individuals treated with four or more glucose-lowering agents; after combining the three aforementioned exclusions; after excluding individuals without linked hospital inpatient data (for both the primary outcome and secondary outcomes of major adverse cardiovascular events and hospitalisation for heart failure); and after including individuals with missing baseline uACR, using multiple-imputed uACR values (described later); and after fitting an interaction term by tertiles of percentage weight change at 12 months after initiation (or if unavailable at 6 months). Finally, we compared risks of the primary and secondary outcomes between GLP-1 receptor agonist initiation and DPP-4 inhibitor initiation and sulfonylurea initiation, and also DPP-4 inhibitor initiations versus sulfonylurea initiation, to check the validity of treating these as a single comparator group.

Missing clinical data (IMD quintile 18·4%, BMI 2·1% [either height or weight missing], blood pressure 0·4%, total cholesterol 0·3%, glycated haemoglobin 0·6%, diabetes duration 6·0%, smoking status 0·5%, and previous hospitalisation 18·4%) were imputed 100 times using multivariate imputations with chained equations. Imputation was done under the missing-at-random assumption, which was considered plausible given the comprehensive set of covariates available to account for mechanisms driving missingness. IMD and smoking status were imputed using multinomial regression, BMI was imputed using passive imputation from height and weight after imputation, and all other variables were imputed using predictive mean matching. We carried out all analyses in each individual imputed dataset and pooled the results according to Rubin’s rules.^26^ All analyses were performed using R (version 4.4.1). We followed TARGET reporting guidelines.^27^

### Role of the funding source

The funder of the study had no role in study design, data collection, data analysis, data interpretation, writing of the report, or the decision to submit for publication.

## Results

**Figure 1:**
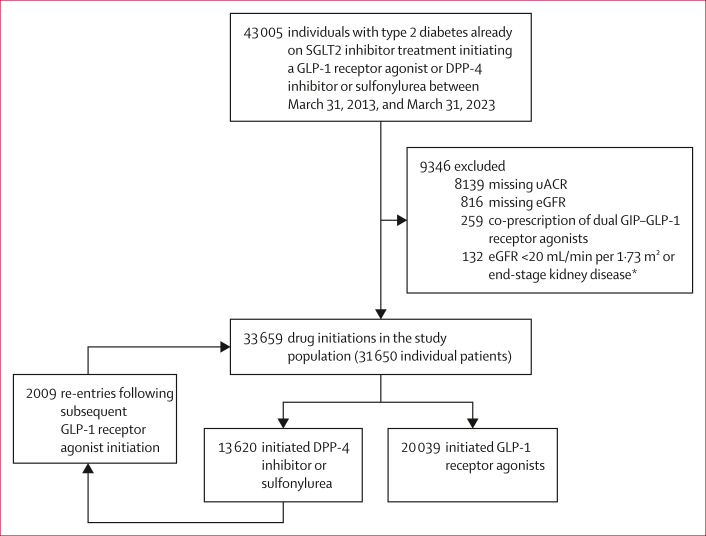
Study population selection eGFR=estimated glomerular filtration rate. uACR=urinary albumin–creatinine ratio. GIP=glucose-dependent insulinotropic polypeptide. ∗End-stage kidney disease is defined as requirement for renal replacement therapy or sustained eGFR of less than 15 mL/min per 1·73 m^2^.

We included 33 659 drug initiations among 31 650 individuals with type 2 diabetes already receiving SGLT2 inhibitor treatment who initiated a GLP-1 receptor agonist (20 039 initiations) or a DPP-4 inhibitor or sulfonylurea (13 620 initiations; figure 1). The median age was 60 years (IQR 53–67), 20 239 (60%) of 33 659 initiations occurred in male individuals and 13 420 (40%) in female individuals, mean eGFR was 92 mL/min per 1·73 m^2^ (SD 18), and median uACR was 1·6 mg/mmol (IQR 0·8–3·4). 8205 (24%) initiations had previous atherosclerotic cardiovascular disease and 2307 (7%) had heart failure. Linked hospital inpatient data were available for 27 401 (82%) initiations. The initiated GLP-1 receptor agonist preparations included dulaglutide (8296 initiations), subcutaneous semaglutide (6312 initiations), oral semaglutide (2680 initiations), liraglutide (1967 initiations), exenatide (533 initiations), and lixisenatide (251 initiations). Before overlap weighting, individuals initiating GLP-1 receptor agonists were more likely to have a higher BMI (standardised mean difference 0·38), a longer duration of diabetes (standardised mean difference 0·38), and to be using four or more glucose-lowering treatments (standardised mean difference 0·60) compared with those initiating DPP-4 inhibitors or sulfonylureas (table), although there was considerable overlap in propensity score distributions (appendix p 20). After overlap weighting, baseline characteristics were well balanced, with all standardised mean differences less than 0·1 (table).

**Table:** Baseline characteristics of each initiation∗ by treatment group, before and after overlap weighting

	Before overlap weighting			After overlap weighting		
	SGLT2 inhibitor plus GLP-1 receptor agonist (n=20 039)	SGLT2 inhibitor plus DPP-4 inhibitor or sulfonylurea (n=13 620)	Standardised mean difference	SGLT2 inhibitor plus GLP-1 receptor agonist (n=20 039)	SGLT2 inhibitor plus DPP-4 inhibitor or sulfonylurea (n=13 620)	Standardised mean difference
Sociodemographic characteristics						
Age, years	59 (53–66)	60 (53–68)	0·076	60 (53–67)	60 (53–67)	<0·01
Sex	··	··	0·040	··	··	<0·01
Male	11 892 (59%)	8347 (61%)	··	12 037 (60%)	8182 (60%)	··
Female	8147 (41%)	5273 (39%)	··	8002 (40%)	5438 (40%)	··
Ethnicity	··	··	0·122	··	··	<0·01
White	16 192 (81%)	10 338 (76%)	··	15 762 (79%)	10 713 (79%)	··
South Asian	2384 (12%)	2108 (15%)	··	2712 (14%)	1844 (14%)	··
Black	761 (4%)	637 (5%)	··	821 (4%)	558 (4%)	··
Other	702 (4%)	537 (4%)	··	744 (4%)	505 (4%)	··
Index of Multiple Deprivation quintile	··	··	0·017	··	··	<0·01
1 (least deprived)	3213 (16%)	2182 (16%)	··	3227 (16%)	2193 (16%)	··
2	3637 (18%)	2436 (18%)	··	3638 (18%)	2473 (18%)	··
3	3736 (19%)	2483 (18%)	··	3702 (18%)	2517 (18%)	··
4	4502 (22%)	3140 (23%)	··	4512 (23%)	3066 (23%)	··
5 (most deprived)	4951 (25%)	3379 (25%)	··	4960 (25%)	3371 (25%)	··
Laboratory and vital signs measurements						
BMI, kg/m^2^	35 (7)	32 (7)	0·38	33 (6)	33 (7)	<0·01
Systolic blood pressure, mm Hg	130 (13)	130 (13)	0·052	130 (13)	130 (13)	<0·01
Total cholesterol, mmol/L	4·3 (1·1)	4·4 (1·1)	0·055	4·4 (1·2)	4·4 (1·2)	<0·01
HbA_1c_, mmol/mol	76 (16)	75 (17)	0·049	75 (16)	75 (17)	<0·01
HbA_1c_, %	9·1% (3·6)	9·0% (3·7)	0·049	9·0% (3·7)	9·0% (3·7)	<0·01
eGFR, mL/min per 1·73 m^2^	93 (18)	92 (19)	0·045	92 (18)	92 (19)	<0·01
eGFR <60 mL/min per 1·73 m^2^	1083 (5%)	893 (7%)	0·049	1031 (5%)	869 (6%)	0·012
uACR, mg/mmol	1·7 (0·9–3·5)	1·5 (0·8–3·2)	0·028	1·7 (0·9–3·3)	1·6 (0·8–3·4)	<0·01
Albuminuria status	··	··	0·041	··	··	<0·01
Normal uACR (<3 mg/mmol)	14 462 (72%)	10 078 (74%)	··	14 682 (73%)	9982 (73%)	··
Low-level uACR (3–30 mg/mmol)	4881 (24%)	2602 (19%)	··	4702 (23%)	3180 (23%)	··
Severely increased uACR (≥30 mg/mmol)	696 (3%)	445 (3%)	··	655 (3%)	458 (3%)	··
Clinical characteristics						
Diabetes duration at treatment initiation, years	10·9 (6·9–15·5)	8·3 (4·9–12·6)	0·38	9·6 (6·0–13·8)	9·2 (5·6–13·9)	<0·01
Smoking status	··	··	0·053	··	··	<0·01
Non-smoker	10 047 (50%)	6983 (51%)	··	10 170 (51%)	6912 (51%)	··
Ex-smoker	7087 (35%)	4500 (33%)	··	6838 (34%)	4648 (34%)	··
Current smoker	2905 (14%)	2137 (16%)	··	3031 (15%)	2060 (15%)	··
Atrial fibrillation	1321 (7%)	928 (7%)	<0·01	1313 (7%)	893 (7%)	<0·01
Atherosclerotic cardiovascular disease	5085 (25%)	3120 (23%)	0·058	4725 (24%)	3212 (24%)	<0·01
Heart failure	1384 (7%)	923 (7%)	<0·01	1341 (7%)	911 (7%)	<0·01
Electronic Frailty Index	0·16 (0·09)	0·15 (0·09)	0·15	0·15 (0·08)	0·15 (0·09)	<0·01
Frailty category†	··	··	0·16	··	··	0·011
Fit (<0·12)	8201 (41%)	6627 (49%)	··	9050 (45%)	6252 (46%)	··
Mild frailty (0·12–0·24)	8347 (42%)	5073 (37%)	··	7962 (40%)	5257 (39%)	··
Moderate frailty (0·24–0·36)	2784 (14%)	1474 (11%)	··	2435 (12%)	1633 (12%)	··
Severe frailty (≥0·36)	707 (4%)	446 (3%)	··	592 (3%)	478 (4%)	··
Hospitalisation in the year before baseline	4403 (22%)	3164 (23%)	0·030	4506 (22%)	3063 (22%)	<0·01
Medications						
Number of current glucose-lowering treatments	··	··	0·60	··	··	<0·01
2	1304 (7%)	1695 (12%)	··	1960 (10%)	1332 (10%)	··
3	10 359 (52%)	9734 (72%)	··	13 304 (66%)	9042 (66%)	··
≥4	8376 (42%)	2191 (16%)	··	4775 (24%)	3246 (24%)	··
On statin	18 323 (91%)	12 058 (89%)	0·10	18 015 (90%)	12 244 (90%)	<0·01
On insulin	2522 (13%)	510 (4%)	0·33	1180 (6%)	802 (6%)	<0·01
On angiotensin-converting enzyme inhibitor or angiotensin-II receptor blocker	14 644 (73%)	9043 (66%)	0·15	13 880 (69%)	9434 (69%)	<0·01
Study period						
Calendar year at baseline	··	··	0·31	··	··	<0·01
2013–16	837 (4%)	1204 (9%)	··	1250 (6%)	850 (6%)	··
2017–18	2071 (10%)	2318 (17%)	··	2766 (14%)	1880 (14%)	··
2019–20	4899 (24%)	3451 (25%)	··	5081 (25%)	3453 (25%)	··
2021–22	10 242 (51%)	5441 (40%)	··	9032 (45%)	6139 (45%)	··
2023	1990 (10%)	1206 (9%)	··	1910 (10%)	1298 (10%)	··

Data are mean (SD), n (%), median (IQR), or standardised mean difference. Weighted characteristics are overlap-weighted values normalised to the original sample size. HbA_1c_=glycated haemoglobin. eGFR=estimated glomerular filtration rate (calculated using the Chronic Kidney Disease Epidemiology Collaboration equation). uACR=urinary albumin–creatinine ratio.

∗ Includes 31 650 unique individuals, of whom 2009 initially started on a DPP-4 inhibitor or sulfonylurea and who were subsequently censored and re-entered the study population upon initiation of a GLP-1 receptor agonist.

† Frailty category is based on the Electronic Frailty Index.^22^

**Figure 2:**
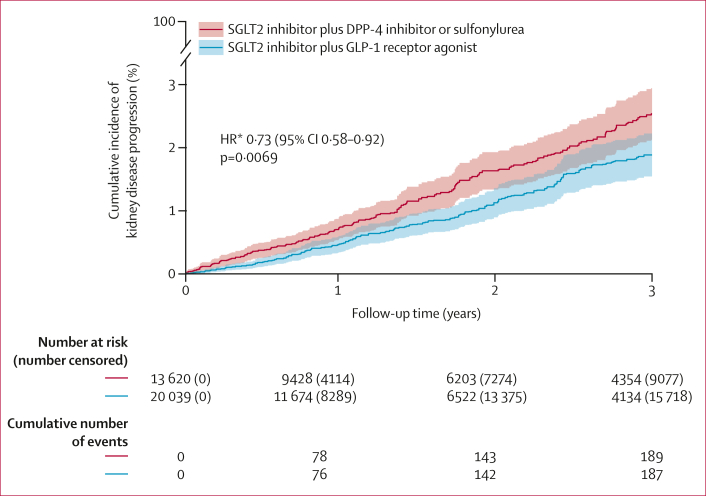
Overlap-weighted cumulative incidence curves of kidney disease progression by treatment group Composite of 40% or more estimated glomerular filtration rate decline, end-stage kidney disease, or death from kidney-related causes. Curves represent overlap-weighted cumulative incidence estimates from Cox proportional hazards models, with shaded 95% CIs. Numbers at risk, cumulative numbers of events, and cumulative numbers censored are unweighted and describe the observed study population. HR=hazard ratio. ∗Double-robust overlap-weighted HR for GLP-1 receptor agonist initiation compared with DPP-4 inhibitor or sulfonylurea initiation.

During a median of 1·4 years (IQR 0·6–3·0) of follow-up, the primary outcome of kidney disease progression (≥40% eGFR decline, end-stage kidney disease, or death from kidney-related causes) occurred in 187 (0·9%) of 20 039 individuals initiating GLP-1 receptor agonists (6·5 events per 1000 person-years) and in 189 (1·4%) of 13 620 initiating DPP-4 inhibitors or sulfonylureas (7·8 events per 1000 person-years). The primary outcome was driven predominantly by sustained eGFR decline of 40% or more (GLP-1 receptor agonists: 172 events [38 identified at end of follow-up]; DPP-4 inhibitors or sulfonylureas: 169 events [34 identified at end of follow-up]; further details on eGFR monitoring are in the appendix [pp 6–7]), followed by end-stage kidney disease (six *vs* 12) and death from kidney-related causes (nine *vs* eight). Initiating GLP-1 receptor agonist treatment was associated with a significantly lower risk of kidney disease progression than was initiating a DPP-4 inhibitor or sulfonylurea (HR 0·73 [95% CI 0·58–0·92], p=0·0069; figure 2). In post-hoc analyses, the corresponding 3-year absolute risk difference was –0·78% (95% CI –1·34 to –0·22), representing a number needed to treat of 128 over 3 years.

The lower hazard of kidney disease progression following GLP-1 receptor agonist initiation was numerically consistent in analyses comparing dulaglutide or subcutaneous semaglutide versus DPP-4 inhibitors or sulfonylureas (HR 0·67 [95% CI 0·51–0·88]) and other GLP-1 receptor agonist preparations versus DPP-4 inhibitors or sulfonylureas, although the latter was not significant (HR 0·74 [0·52–1·05]; for characteristics per group see appendix pp 8–12). Estimates were also consistent in sensitivity analyses extending follow-up to 5 years; censoring individuals after discontinuing treatment; using multivariable adjustment only; using inverse probability of treatment weighting; and accounting for death using overlap-weighted competing risk models (appendix p 21). Similarly, estimates were consistent when excluding individuals initiating GLP-1 receptor agonists following a previous DPP-4 inhibitor or sulfonylurea initiation, those treated with insulin, those treated with four or more glucose-lowering treatments, and combining the three aforementioned exclusions (appendix p 22). Furthermore, estimates were consistent when excluding those without linked hospital inpatient data, and when including those with missing baseline uACR (appendix p 22). There was no evidence of interaction across tertiles of percentage weight loss within 12 months after initiation, although the primary outcome was no longer significant in this analysis (median 3·5% [IQR 0·6 to 6·9] weight loss following GLP-1 receptor agonist initiation versus 0·1% [–2·4 to 3·1] following DPP-4 inhibitor or sulfonylurea initiation; p=0·59 for interaction; appendix p 13). Finally, estimates were generally consistent when comparing GLP-1 receptor agonist initiation with DPP-4 inhibitor initiation alone (HR 0·70 [0·52–0·93]) and sulfonylurea initiation alone (HR 0·77 [0·56–1·07]), although the latter was not significant (appendix pp 23–24). When comparing DPP-4 inhibitor initiation (8481 initiations) with sulfonylurea initiation (5139 initiations; characteristics of the analysis population are shown in the appendix [pp 14–18]), we observed similar risks of kidney disease progression (appendix p 25).

**Figure 3:**
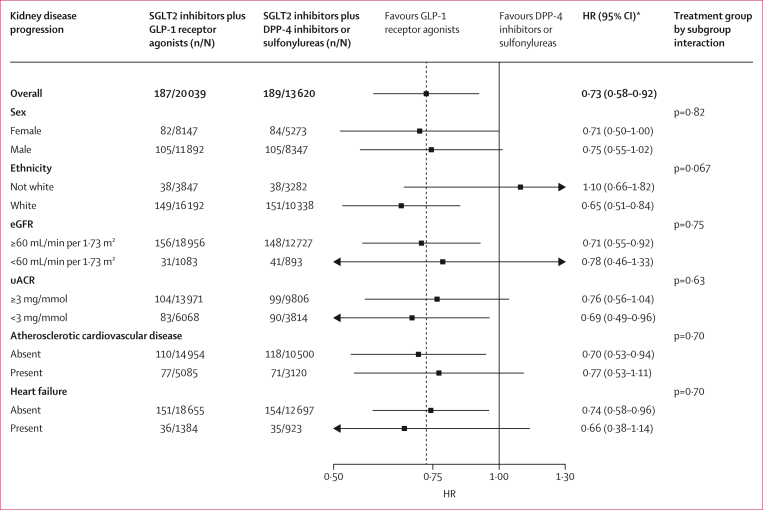
Forest plot of double-robust overlap-weighted HRs for kidney disease progression Composite of 40% or more eGFR decline, end-stage kidney disease, or death from kidney-related causes for GLP-1 receptor agonist initiation compared with DPP-4 inhibitor or sulfonylurea initiation across subgroups defined by sex, ethnicity, eGFR, uACR, previous atherosclerotic cardiovascular disease, and heart failure. p values represent tests for subgroup-level interaction terms. eGFR=estimated glomerular filtration rate. HR=hazard ratio. uACR=urine albumin–creatinine ratio. ∗Double-robust overlap-weighted HRs.

**Figure 4:**
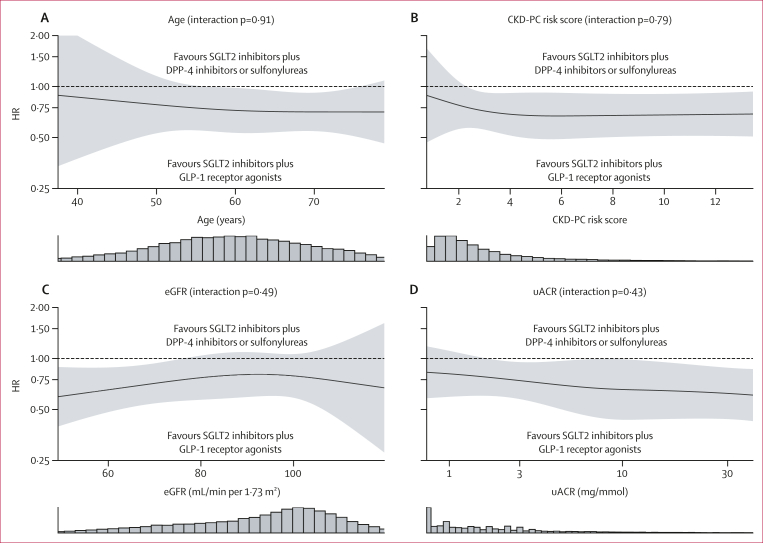
Spline plots of double-robust overlap-weighted HRs for kidney disease progression Composite of 40% or more eGFR decline, end-stage kidney disease, or death from kidney-related causes for GLP-1 receptor agonist initiation compared with DPP-4 inhibitor or sulfonylurea initiation across the continuous spectrums of age (A), predicted 3-year risk of 40% or more eGFR decline or end-stage kidney disease estimated using the CKD-PC risk score (B), eGFR (C), and uACR (D). Continuous non-linear interactions were modelled using restricted cubic splines with three knots. Histograms show the underlying distribution of each feature. p values represent tests for the total linear and non-linear interaction terms. Horizontal splines and non-significant p values indicate a consistent association across the spectrum of the respective feature. CKD-PC=Chronic Kidney Disease Prognosis Consortium. eGFR=estimated glomerular filtration rate. HR=hazard ratio. uACR=urine albumin–creatinine ratio.

The lower risk of kidney disease progression with GLP-1 receptor agonist treatment was generally consistent across subgroups defined by sex, ethnicity, eGFR, uACR, previous atherosclerotic cardiovascular disease, and heart failure (p>0·05 for all interactions; figure 3). The association was also generally consistent across the continuous spectrums of age, eGFR, uACR, and predicted 3-year risk of 40% or more eGFR decline or end-stage kidney disease estimated with the CKD-PC risk score (p>0·05 for all interactions; figure 4).

Initiating GLP-1 receptor agonists was associated with a borderline lower risk of major adverse cardiovascular events (HR 0·89 [95% CI 0·80–1·00]) and hospitalisation for heart failure (HR 0·86 [0·76–0·98]) compared with initiation of DPP-4 inhibitors or sulfonylureas (appendix p 19). When excluding individuals without linked hospital inpatient data, these associations were numerically consistent but no longer significant (HR 0·89 [0·79–1·00] for major adverse cardiovascular events and 0·91 [0·79–1·05] for hospitalisation for heart failure). To assess potential harms, we evaluated prespecified safety outcomes. We found no evidence of an increased risk of acute pancreatitis (32 [0·2%] of 19 727 individuals with no recorded history of pancreatitis who initiated a GLP-1 receptor agonist *vs* 25 [0·2%] of 13 305 who initiated a DPP-4 inhibitor or sulfonylurea; HR 0·94 [0·52–1·70]) or incident diabetic retinopathy (1097 [10·1%] of 10 844 individuals with no recorded history of diabetic retinopathy who initiated a GLP-1 receptor agonist *vs* 936 [10·7%] of 8719 who initiated a DPP-4 inhibitor or sulfonylurea; HR 1·07 [0·97–1·18]) with GLP-1 receptor agonist initiation compared with DPP-4 inhibitors or sulfonylureas. The neutral control outcome, lower limb fracture, showed no association with GLP-1 receptor agonist initiation compared with DPP-4 inhibitors or sulfonylureas (HR 0·92 [0·65–1·30]; appendix p 19). Sensitivity analyses comparing GLP-1 receptor agonist initiation with DPP-4 inhibitor initiation alone and with sulfonylurea initiation alone, and when comparing DPP-4 inhibitor initiation with sulfonylurea initiation, resulted in generally similar secondary, safety, and neutral outcome estimates (appendix pp 23–25).

## Discussion

We found that, in individuals with type 2 diabetes already receiving SGLT2 inhibitor treatment, initiating GLP-1 receptor agonists is associated with a significantly lower risk of kidney disease progression compared with initiating comparator drugs (DPP-4 inhibitors or sulfonylureas). Our analyses reveal no evidence of effect modification by eGFR, uACR, or predicted risk of kidney disease progression, suggesting that adding GLP-1 receptor agonists to SGLT2 inhibitor treatment could provide a consistent relative risk reduction across the spectrum of kidney disease severity.

The kidney benefits associated with GLP-1 receptor agonist initiation in individuals already receiving SGLT2 inhibitor treatment align with those seen in large randomised trials evaluating GLP-1 receptor agonist treatment versus placebo. In the FLOW trial, which randomly allocated 3533 participants with type 2 diabetes and chronic kidney disease to subcutaneous semaglutide or placebo, semaglutide reduced the risk of a kidney-specific composite outcome (≥50% eGFR decline, end-stage kidney disease, or death from kidney-related causes) by 21% (HR 0·79 [95% CI 0·66–0·94]).^5^ Similarly, in post-hoc analyses of the REWIND trial, which randomly allocated 9901 patients with type 2 diabetes and cardiovascular disease or cardiovascular risk factors to dulaglutide or placebo, dulaglutide lowered the risk of the kidney composite outcome (≥40% eGFR decline, end-stage kidney disease, or death from kidney-related causes) by 25% (HR 0·75 [0·62–0·92]).^25^ Because these trials primarily compared GLP-1 receptor agonist treatment with placebo and were not designed to evaluate its efficacy among individuals already receiving SGLT2 inhibitor treatment, our findings complement the evidence base by suggesting that a similar relative risk reduction is preserved when adding GLP-1 receptor agonists to SGLT2 inhibitor treatment in real-world clinical practice compared with adding a DPP-4 inhibitor or sulfonylurea.

An additive kidney-protective effect with combined SGLT2 inhibitor and GLP-1 receptor agonist treatment is biologically plausible given their distinct mechanisms of action. SGLT2 inhibitors reduce glomerular hyperfiltration,^28^ whereas GLP-1 receptor agonists have anti-inflammatory and antioxidant effects.^29^ Although GLP-1 receptor agonist-associated loss of muscle mass could theoretically lead to an apparent improvement in creatinine-based eGFR, we found no interaction with weight loss. Moreover, in the FLOW trial, semaglutide similarly preserved kidney function when eGFR was estimated from either creatinine or cystatin C,^5^ suggesting that changes in muscle mass do not explain the observed association.

Our findings regarding cardiovascular and safety outcomes align with existing literature, reinforcing the validity of our kidney outcome findings. The lower risks of major adverse cardiovascular events and hospitalisation for heart failure observed with GLP-1 receptor agonist initiation in individuals already receiving SGLT2 inhibitor treatment are consistent with previous clinical trials comparing GLP-1 receptor agonists versus placebo^2^ as well as observational studies comparing combined GLP-1 receptor agonist–SGLT2 inhibitor treatment with SGLT2 inhibitors alone.^7^, ^8^, ^9^^,^^11^ We did not observe an increased risk of acute pancreatitis or incident diabetic retinopathy following GLP-1 receptor agonist initiation, in keeping with findings from a recent trial meta-analysis.^2^

Our study has several strengths. To our knowledge, this is the first large-scale observational study to specifically evaluate kidney outcomes after initiation of GLP-1 receptor agonist treatment in individuals with type 2 diabetes already receiving SGLT2 inhibitor treatment. We followed a target trial emulation framework, specifying eligibility criteria, treatment strategies, and follow-up to mirror a pragmatic randomised trial. This approach minimised bias related to treatment timing and selection. For example, by comparing individuals who all required additional glucose-lowering treatment on top of SGLT2 inhibitors, differences due to stage of diabetes progression were reduced. We used a standardised kidney composite outcome consistent with clinical trial and regulatory definitions,^18^ ensuring comparability with existing evidence. Within the study population, we observed a substantial degree of overlap in treatment probability between treatment groups, supporting the positivity assumption that individuals in both groups theoretically could have received either treatment. Furthermore, we used a double-robust approach, which helps to maintain valid estimates even if either the propensity score or the outcome model were misspecified.^13^ Extensive sensitivity analyses supported the robustness of our findings, and we observed internal consistency across secondary outcomes and the control outcome. By evaluating relative treatment effects across strata of cardiovascular disease, heart failure, and kidney disease severity in real-world data, we ensured that our findings are generalisable across the broad spectrum of individuals with type 2 diabetes treated with SGLT2 inhibitors in routine clinical practice in the UK.

Several limitations should be acknowledged. First, as with all observational studies, residual confounding cannot be fully ruled out. Although unmeasured factors, such as patient preference, pre-baseline weight trajectory, or frailty not fully captured by the Electronic Frailty Index might have influenced treatment selection, the absence of association with a negative control outcome nevertheless reduces the likelihood of major residual bias affecting our results. Second, our primary outcome included cause-specific mortality, which can be subject to misclassification. Nevertheless, competing risk analysis yielded consistent results with our primary analysis, suggesting that mortality did not meaningfully bias estimates. Third, the study cohort was predominantly White, precluding precise assessment of heterogeneity across specific ethnic groups. Although we did not find evidence of a significant interaction by ethnicity, it remains unclear whether directional differences reflect true effect modification or imprecision. Nevertheless, our findings remain potentially applicable to wider populations meeting our target trial eligibility criteria within similar universal, primary-care-led health systems. Fourth, the median follow-up was relatively short, which restricted our ability to assess long-term kidney protection. Fifth, we did not assess treatment discontinuation, tolerability, or dose-specific effects. Sixth, we combined DPP-4 inhibitors and sulfonylureas into a single comparator group to increase statistical power for heterogeneity analyses. Although this grouping is supported by evidence that neither class alters kidney disease progression, it creates a comparator population with heterogeneous clinical profiles (eg, varying risks of hypoglycaemia or weight gain). This could theoretically restrict the transferability of our findings to specific clinical scenarios where these metabolic side-effects drive treatment choice. Nevertheless, our sensitivity analyses showed consistent benefits of GLP-1 receptor agonists regardless of whether they were compared with DPP-4 inhibitors or sulfonylureas separately, suggesting that this heterogeneity did not meaningfully confound the primary kidney outcome results. Seventh, novel treatments, such as finerenone, were not in use in the UK during the study period, and subgroup analysis by individual GLP-1 receptor agonist preparation was not viable due to small sample sizes. Further research is needed to compare newer treatments and individual GLP-1 receptor agonist preparations, and to establish optimal treatment sequencing and combination strategies. Finally, dual-drug prescriptions increase polypharmacy and costs, which may limit transferability to all clinical settings. This necessitates a targeted approach prioritising individuals with the highest potential clinical benefit.

Taken together, our findings support a potential role for adding GLP-1 receptor agonists to SGLT2 inhibitor treatment for kidney protection in individuals with type 2 diabetes. Although current international type 2 diabetes guidelines primarily recommend adding GLP-1 receptor agonists to SGLT2 inhibitors for cardiovascular protection,^4^ our results support extending this rationale to kidney protection. Because chronic kidney disease exists along a continuum, treatment decisions should be guided by absolute rather than relative risk reductions. Post-hoc analyses showed a population-average 3-year absolute risk difference of 0·78%. However, the consistent hazard reductions by eGFR, uACR, and predicted baseline risk we observed suggest that clinical benefit could be maximised by prioritising treatment for those at highest baseline risk of kidney disease progression. Specifically, individual-level absolute benefit could be predicted by integrating trial-derived relative risk reductions with validated tools such as the CKD-PC risk score,^30^ a validated framework that allows clinicians to weigh the magnitude of kidney protection against considerations of treatment burden and cost.

In summary, initiation of GLP-1 receptor agonist treatment in individuals with type 2 diabetes already receiving SGLT2 inhibitor treatment was associated with a lower hazard of kidney disease progression than initiation of a DPP-4 inhibitor or sulfonylurea, consistent with an additive protective effect. The generally consistent benefit across the spectrum of kidney disease severity supports a potential role for GLP-1 receptor agonists as an add-on to SGLT2 inhibitor treatment for kidney protection, particularly among those at highest absolute risk. Further prospective data would be valuable to confirm these findings.

## Data sharing

All CPRD data are available by application to the CPRD Independent Scientific Advisory Committee (https://cprd.com/data-access). All code to develop the cohort used in this study is available at https://github.com/Exeter-Diabetes/CPRD-Cohort-scripts/tree/main/03-Treatment-response-(MASTERMIND). Although a formal study protocol was not prospectively registered, all code to reproduce the analyses in this study is available at https://github.com/Exeter-Diabetes/CPRD-Thijs-GLP1-KF.

## Declaration of interests

JMD and PC report support by a Wellcome Trust Early Career award (227070/Z/23/Z). JMD and BMS report support by the National Institute for Health and Care Research (NIHR) Exeter Clinical Research Facility. AGJ reports institutional research funding from the UK Medical Research Council, NIHR, Diabetes UK, Breakthrough Type 1 Diabetes, the Novo Nordisk Foundation, and the European Foundation for the Study of Diabetes; and support for travel or accommodation for speaking at meetings from Diabetes UK, the European Association for the Study of Diabetes, the Society of Endocrinology, Diabetes and Metabolism of South Africa, and NIHR. RAO reports consulting income from Sanofi; honoraria from Sanofi, Janssen, and Novo Nordisk; royalties from Randox for commercial use of a type 1 diabetes genetic risk score array; grants from Randox and Sanofi; and research funding from Breakthrough Type 1 Diabetes, Diabetes UK, The Leona M and Larry B Helmsley Charitable Trust, and Randox. BMS reports grants from and participation on a data safety monitoring board or advisory board with Diabetes UK. All other authors declare no competing interests.
