# Supplementary material for: Kidney outcomes with GLP-1 receptor agonists in people with type 2 diabetes already receiving SGLT2 inhibitors: a target trial emulation study using UK primary care data
Source: Lancet Prim Care. 2026 Apr;2(4):None. doi: 10.1016/j.lanprc.2026.100139 (PMC13153036; doi:10.1016/j.lanprc.2026.100139)
Supplement: Supplementary appendix [file mmc1.pdf]

THE LANCET
Primary Care

Supplementary appendix

This appendix formed part of the original submission and has been peer reviewed. We post it as supplied by the authors.

Supplement to: Jansz TT, McGovern AP, Young KG, et al. Kidney outcomes with GLP-1 receptor agonists in people with type 2 diabetes already receiving SGLT2 inhibitors: a target trial emulation study using UK primary care data. *Lancet Prim Care* 2026. https://doi.org/10.1016/j.lanprc.2026.100139

APPENDIX

Table of contents	Page
Supplemental Table 1	2
Supplemental Table 2	6
Supplemental Table 3	8
Supplemental Table 4	13
Supplemental Table 5	14
Supplemental Table 6	19
Supplemental Figure 1	20
Supplemental Figure 2	21
Supplemental Figure 3	22
Supplemental Figure 4	23

Supplemental Table 1. Target trial emulation specification.

Protocol components	Target trial	Emulation
Eligibility criteria	Inclusion:<br>- Type 2 diabetes<br>- Already receiving SGLT2i treatment<br>- No previous prescriptions for GLP1-RA treatment.<br><br>Exclusion:<br>- eGFR <20 ml/min per $1.73m^2$ or end-stage kidney disease (requirement for renal replacement therapy or sustained eGFR <15 ml/min per $1.73m^2$).<br>- Use of dual glucose-dependent insulinotropic polypeptide and glucagon-like peptide-1 receptor agonists.	Inclusion<br>- Type 2 diabetes<br>- Already receiving SGLT2i treatment<br>- No previous prescriptions for GLP1-RA treatment.<br>- Receiving first-observed prescription for GLP1-RA, DPP4i, or SU.<br><br>Exclusion:<br>- eGFR <20 ml/min per $1.73m^2$ or end-stage kidney disease (requirement for renal replacement therapy or sustained eGFR <15 ml/min per $1.73m^2$).<br>- Missing eGFR or urinary albumin creatinine ratio.<br>- Use of dual glucose-dependent insulinotropic polypeptide and glucagon-like peptide-1 receptor agonists.<br>- Less than 91 days of registration data before treatment initiation.
Treatment assignment	Random unblinded assignment to one of the two treatment arms (GLP1-RA vs DPP4i/SU).	We assume random assignment to one of the two treatment arms (GLP1-RA vs DPP4i/SU) by applying double-robust overlap weighting based on predefined covariates.
Treatment initiation	Initiation of only one glucose-lowering medication (GLP1-RA, DPP4i, or SU) at the time of treatment assignment.	We consider the date of a first-observed prescription for one glucose-lowering medication (GLP1-RA, DPP4i, or SU) as date of treatment initiation.

Treatment strategy	Treatment initiation is defined as a prescription issued for GLP1-RA or DPP4i/SU by primary care practice. For the per-protocol analysis, continuation is defined as no gap in prescriptions exceeding 183 days. Further additional glucose-lowering treatment with DPP4i or SU is allowed as needed. Subjects in the DPP4i/SU arm are not allowed to initiate GLP1-RA treatment.	Treatment initiation is defined as a first-observed prescription for GLP1-RA or DPP4i/SU as identified from primary care records (from the Clinical Practice Research Datalink). For the per-protocol analysis, continuation is defined as no gap in prescriptions exceeding 183 days. Further additional glucose-lowering treatment with DPP4i or SU is allowed as needed. Subjects in the DPP4i/SU arm are censored upon initiation of a GLP1-RA; they may subsequently re-enter the study in the GLP1-RA arm (contributing non-overlapping observation periods).
Follow-up period	Follow-up starts at treatment initiation. Subjects are followed up until the earliest of: occurrence of outcome, a maximum of 3 years, or March 31, 2023.	Follow-up starts at treatment initiation. Subjects are followed up until the earliest of: occurrence of outcome, a maximum of 3 years, or March 31, 2023. Subjects in the DPP4i/SU arm are censored if initiating GLP1-RA.
Outcomes	Primary outcome:<br>- Kidney disease progression, defined as a composite of a sustained ≥40% decline in eGFR, end-stage kidney disease (requirement for renal replacement therapy or sustained eGFR <15 ml/min per 1.73m^2), or death from kidney-related causes.<br>Secondary outcomes:<br>- Major adverse cardiovascular events (myocardial infarction, stroke, or death from cardiovascular causes).<br>- Hospitalisation for heart failure.<br>- Incident diabetic retinopathy.<br>- Acute pancreatitis.	Primary outcome:<br>- Kidney disease progression, defined as a composite of a sustained ≥40% decline in eGFR, end-stage kidney disease (requirement for renal replacement therapy or sustained eGFR <15 ml/min per 1.73m^2), or death from kidney-related causes.<br>Secondary outcomes:<br>- Major adverse cardiovascular events (myocardial infarction, stroke, or death from cardiovascular causes).<br>- Hospitalisation for heart failure.<br>- Incident diabetic retinopathy.<br>- Acute pancreatitis.<br>Neutral control outcome:<br>- Lower limb fracture.

Causal contrasts of interest	Intention-to-treat and per-protocol estimands.	The primary analysis uses a pragmatic intention-to-treat estimand. Subjects in the comparator arm are censored if initiating GLP1-RA but are allowed to re-enter the study in the GLP1-RA arm (contributing non-overlapping observation periods). Initiation of additional comparator agents (DPP4i/SU) does not trigger censoring.<br>As sensitivity analyses, we estimate<br>- a strict intention-to-treat estimand, where subjects in the comparator arm initiating GLP1-RA were censored and were not allowed to re-enter the study.<br>- a per-protocol estimand, censoring subjects if the assigned treatment is discontinued (defined by a gap in prescriptions exceeding a 183-day grace period).
Analysis plan	- Cox proportional hazard models.<br>- Subgroup analyses by strata of sex, ethnicity, history of atherosclerotic cardiovascular disease, and heart failure.	- Double-robust Cox proportional hazards models (concurrent multivariable adjustment and overlap weighting).<br>- Subgroup analyses by strata of sex, ethnicity, history of atherosclerotic cardiovascular disease, and heart failure.<br>- Assessment of continuous effect modification by age, eGFR, uACR, and predicted 3-year risk of kidney disease progression (CKD-PC score) using restricted cubic spline model<br>- Sensitivity analyses with different analysis approaches: extending follow-up up to 5 years, per-protocol analysis; multivariable adjustment alone, inverse probability of treatment weighting, and Fine-Gray competing risk models accounting for death (overlap-weighted).<br>- Sensitivity analyses excluding and including specific patient subgroups: those initiating GLP1-RA following a previous DPP4i/SU episode, those treated with insulin, those treated with >4

		glucose-lowering treatments, those meeting any of the 3 previous criteria, those without linkage to hospital inpatient data; and including individuals with missing baseline uACR, using imputed values.<br>- Sensitivity analyses with DPP4i and SU treated as separate treatment arms.

Abbreviations: SGLT2i sodium-glucose cotransporter-2 inhibitors, GLP1-RA glucagon-like peptide-1 receptor agonists, DPP4i dipeptidyl peptidase-4 inhibitors, SU sulfonylureas, eGFR estimated glomerular filtration rate (calculated using the Chronic Kidney Disease Epidemiology Collaboration equation), BMI body mass index, uACR urinary albumin/creatinine ratio, CKD-PC Chronic Kidney Disease Prognosis Consortium.

Supplemental Table 2. eGFR monitoring frequency by treatment arm, before and after overlap weighting.

	Before overlap weighting			After overlap weighting		
	SGLT2i + GLP1-RA (n=20,039)	SGLT2i + DPP4i/SU (n=13,620)	SMD	SGLT2i + GLP1-RA (n=20,039)	SGLT2i + DPP4i/SU (n=13,620)	SMD
Number of eGFR readings in the year before baseline (median, IQR)	2 [2, 3]	2 [2, 3]	0.025	2 [2, 3]	2 [2, 3]	0.051
Number of eGFR readings in the year before baseline (n, %)			0.043			0.014
1	4,097 (20%)	3,011 (22%)		4,027 (20%)	3,085 (23%)	
2	7,857 (39%)	5,311 (39%)		7,823 (39%)	5,335 (39%)	
3	4,770 (24%)	3,133 (23%)		4,813 (24%)	3,076 (23%)	
≥4	3,315 (17%)	2,165 (16%)		3,376 (17%)	2,124 (16%)	
Total follow-up time post-initiation (mean, SD)	1.4 (1.1)	1.8 (1.1)	0.31	1.6 (1.1)	1.7 (1.1)	0.10
Number of eGFR readings in the year after baseline (median, IQR)	1 [1, 2]	1 [0, 2]	<0.01	1 [1, 2]	1 [0, 2]	0.019
Number of eGFR readings in the year after baseline (n, %)			0.046			0.015
0	4,949 (25%)	3,549 (26%)		4,912 (25%)	3,607 (26%)	
1	7,975 (40%)	5,405 (40%)		8,015 (40%)	5,399 (40%)	
2	4,496 (22%)	2,884 (21%)		4,491 (22%)	2,846 (21%)	
3	1,666 (8%)	1,071 (8%)		1,664 (8%)	1,066 (8%)	
≥4	953 (5%)	711 (5%)		957 (5%)	702 (5%)	

Abbreviations: SGLT2i sodium-glucose cotransporter-2 inhibitors, GLP1-RA glucagon-like peptide-1 receptor agonists, DPP4i dipeptidyl peptidase-4 inhibitors, SU sulfonylureas, SMD standardised mean difference, SD standard deviation, IQR interquartile range, eGFR estimated glomerular filtration rate (calculated using the Chronic Kidney Disease Epidemiology Collaboration equation).

Weighted characteristics are overlap-weighted values normalised to the original sample size.

Supplemental Table 3. Baseline characteristics at initiation of dulaglutide/subcutaneous semaglutide, other GLP1-RA preparations, and DPP4i/SU, before and after overlap weighting.

	Before overlap weighting				After overlap weighting			
	SGLT2i + dulaglutide / subcutaneous semaglutide (n=14,608)	SGLT2i + other GLP1-RA (n=5,431)	SGLT2i + DPP4i/SU (n=13,620)	SMD	SGLT2i + dulaglutide / subcutaneous semaglutide (n=14,608)	SGLT2i + other GLP1-RA (n=5,431)	SGLT2i + DPP4i/SU (n=13,620)	SMD
Sociodemographic characteristics								
Age, years (mean, SD)	59 (11)	59 (10)	60 (11)	0.052	59 (10)	59 (10)	59 (11)	0.011
Sex (n, %)				0.026				0.013
Male	8,669 (59%)	3,223 (59%)	8,347 (61%)		8,712 (60%)	3,266 (60%)	8,016 (59%)	
Female	5,939 (41%)	2,208 (41%)	5,273 (39%)		5,896 (40%)	2,165 (40%)	5,604 (41%)	
Ethnicity (n, %)				0.092				<0.01
White	11,834 (81%)	4,358 (80%)	10,338 (76%)		11,583 (79%)	4,290 (79%)	10,877 (80%)	
South Asian	1,741 (12%)	643 (12%)	2,108 (15%)		1,876 (13%)	694 (13%)	1,700 (12%)	
Black	545 (4%)	216 (4%)	637 (5%)		596 (4%)	230 (4%)	544 (4%)	
Other	488 (3%)	214 (4%)	537 (4%)		553 (4%)	217 (4%)	499 (4%)	
Index of multiple deprivation, quintiles (n, %)				0.040				<0.01
1 (least deprived)	2,398 (16%)	815 (15%)	2,182 (16%)		2,305 (16%)	842 (15%)	2,108 (15%)	

2	2,669 (18%)	968 (18%)	2,436 (18%)		2,591 (18%)	970 (18%)	2,470 (18%)	
3	2,728 (19%)	1,008 (19%)	2,483 (18%)		2,735 (19%)	1,033 (19%)	2,543 (19%)	
4	3,298 (23%)	1,204 (22%)	3,140 (23%)		3,275 (23%)	1,224 (23%)	2,987 (22%)	
5 (most deprived)	3,515 (24%)	1,436 (26%)	3,379 (25%)		3,702 (25%)	1,362 (25%)	3,512 (26%)	
Laboratory and vital signs measurements								
BMI, kg/m^2 (mean, SD)	35 (7)	35 (7)	32 (7)	0.26	34 (6)	34 (6)	34 (8)	0.088
Systolic blood pressure, mmHg (mean, SD)	130 (13)	130 (13)	130 (13)	0.042	130 (13)	130 (13)	130 (13)	0.010
Total cholesterol, mmol/L (mean, SD)	4.3 (1.1)	4.3 (1.1)	4.4 (1.2)	0.040	4.3 (1.2)	4.3 (1.1)	4.4 (1.2)	0.021
HbA_{1c}, mmol/mol (mean, SD)	76 (16)	76 (16)	75 (17)	0.035	75 (16)	75 (16)	76 (17)	0.021
HbA_{1c}, % (mean, SD)	9.1 (3.6)	9.1 (3.6)	9.0 (3.7)	0.035	9.1 (3.7)	9.0 (3.6)	9.1 (3.7)	0.021
eGFR, mL/min per 1.73m^2 (mean, SD)	93 (18)	93 (18)	92 (19)	0.031	93 (18)	93 (18)	93 (19)	<0.01
eGFR <60 mL/min per 1.73m^2 (n, %)	780 (5%)	303 (6%)	893 (7%)	0.034	758 (5%)	302 (6%)	912 (7%)	0.015
uACR, mg/mmol (median, IQR)	1.7 [0.9, 3.4]	1.7 [0.9, 3.5]	1.5 [0.8, 3.2]	0.034	1.7 [0.9, 3.4]	1.7 [0.9, 3.4]	1.6 [0.9, 3.5]	0.010
Albuminuria status (n, %)				0.038				<0.01
Normal uACR (<3mg/mmol)	10,570 (72%)	3,892 (72%)	10,078 (74%)		10,582 (72%)	3,934 (72%)	9,832 (72%)	

Low-level uACR (3-30mg/mmol)	3,546 (24%)	1,335 (25%)	3,097 (23%)		3,512 (24%)	1,314 (24%)	3,277 (24%)	
Severely increased uACR (≥30mg/mmol)	492 (3%)	204 (4%)	445 (3%)		514 (4%)	183 (3%)	511 (4%)	
Clinical characteristics								
Diabetes duration at treatment initiation, years (median, IQR)	10.9 [6.9, 15.6]	10.8 [6.8, 15.3]	8.3 [4.9, 12.6]	0.26	10.1 [6.4, 14.6]	10.2 [6.5, 14.6]	10.1 [6.1, 15.1]	0.043
Smoking status (n, %)				0.037				<0.01
Non-smoker	7,316 (50%)	2,731 (50%)	6,983 (51%)		7,407 (61%)	2,737 (50%)	6,915 (51%)	
Ex-smoker	5,179 (35%)	1,908 (35%)	4,500 (33%)		5,018 (34%)	1,891 (35%)	4,703 (35%)	
Current smoker	2,113 (14%)	792 (15%)	2,137 (16%)		2,183 (15%)	803 (15%)	2,002 (15%)	
Atrial fibrillation (n, %)	970 (7%)	351 (6%)	928 (7%)	<0.01	950 (7%)	358 (7%)	902 (7%)	<0.01
Atherosclerotic cardiovascular disease (n, %)	3,725 (25%)	1,360 (25%)	3,120 (23%)	0.040	3,493 (24%)	1,319 (24%)	3,309 (24%)	<0.01
Heart failure (n, %)	1,023 (7%)	361 (7%)	923 (7%)	<0.01	983 (7%)	364 (7%)	914 (7%)	<0.01
Electronic frailty index (mean, SD)	0.16 (0.09)	0.16 (0.09)	0.15 (0.09)	0.10	0.15 (0.09)	0.15 (0.08)	0.16 (0.09)	0.020
Frailty category* (n, %)				0.12				0.018
Fit (<0.12)	5,983 (41%)	2,218 (41%)	6,627 (49%)		6,346 (43%)	2,311 (43%)	5,895 (43%)	
Mild frailty (0.12-0.24)	6,060 (41%)	2,287 (42%)	5,073 (37%)		5,899 (40%)	2,252 (41%)	5,398 (40%)	
Moderate frailty (0.24-0.36)	2,036 (14%)	748 (14%)	1,474 (11%)		1,882 (13%)	710 (13%)	1,805 (13%)	

Severe frailty (≥0.36)	529 (4%)	178 (3%)	446 (3%)		481 (3%)	158 (3%)	522 (4%)	
Hospitalisation in the year before baseline (n, %)	3,220 (22%)	1,184 (22%)	3,164 (23%)	0.022	3,201 (22%)	1,194 (22%)	3,037 (22%)	<0.01
Medications								
Number of current glucose-lowering treatments (n, %)				0.42				<0.01
2	964 (7%)	340 (6%)	1,695 (12%)		1,134 (8%)	427 (8%)	1,073 (8%)	
3	7,609 (52%)	2,750 (51%)	9,734 (72%)		8,735 (60%)	3,219 (59%)	8,052 (59%)	
4+	6,035 (41%)	2,341 (43%)	2,191 (16%)		4,739 (32%)	1,785 (33%)	4,495 (33%)	
On statin (n, %)	13,358 (91%)	4,965 (91%)	12,058 (89%)	0.065	13,265 (91%)	4,932 (91%)	12,346 (91%)	<0.01
On insulin (n, %)	1,844 (13%)	678 (12%)	510 (4%)	0.22	1,192 (8%)	476 (9%)	1,237 (9%)	<0.01
On ACE inhibitor or angiotensin-II receptor blocker (n, %)	10,681 (73%)	3,963 (73%)	9,043 (66%)	0.10	10,358 (71%)	3,841 (71%)	9,701 (71%)	<0.01
Study period								
Calendar year at baseline (n, %)				0.11				0.015
2013-2016	207 (1%)	630 (12%)	1,204 (9%)		771 (5%)	282 (5%)	760 (6%)	
2017-2018	1,222 (8%)	849 (16%)	2,318 (17%)		2,292 (16%)	797 (15%)	2,132 (16%)	
2019-2020	4,219 (29%)	680 (13%)	3,451 (25%)		2,759 (19%)	994 (18%)	2,539 (19%)	
2021-2022	7,974 (55%)	2,268 (42%)	5,441 (40%)		6,960 (48%)	2,670 (49%)	6,501 (48%)	
2023	986 (7%)	1,004 (18%)	1,206 (9%)		1,826 (13%)	688 (13%)	1,688 (12%)	

Abbreviations: SGLT2i sodium-glucose cotransporter-2 inhibitors, GLP1-RA glucagon-like peptide-1 receptor agonists, DPP4i dipeptidyl peptidase-4 inhibitors, SU sulfonylureas, SMD standardised mean difference, SD standard deviation, IQR interquartile range, BMI body mass index, eGFR estimated glomerular filtration rate (calculated using the Chronic Kidney Disease Epidemiology Collaboration equation), uACR urinary albumin/creatinine ratio.

Weighted characteristics are overlap-weighted values normalised to the original sample size.

*Frailty category based on the electronic frailty index.

Supplemental Table 4. Event numbers and HRs of the primary outcome (≥40% eGFR decline, ESKD, or death from kidney-related causes) by tertiles of percent weight loss within 12 months* per treatment arm.

	SGLT2i + GLP1-RA		SGLT2i + DPP4i/SU		
Weight loss tertile†	Number of individuals at risk	Number of events	Number of individuals at risk	Number of events	HR‡ (95%CI)
Tertile 1 (<0.1%)	2,528	24	3,705	44	0.86 (0.47, 1.58)
Tertile 2 (0.1 – 4.3%)	3,857	24	2,360	31	0.56 (0.31, 1.02)
Tertile 3 (≥4.3%)	4,891	35	1,360	17	0.76 (0.39, 1.46)

Abbreviations: SGLT2i sodium-glucose cotransporter-2 inhibitors, GLP1-RA glucagon-like peptide-1 receptor agonists, DPP4i dipeptidyl peptidase-4 inhibitors, SU sulfonylureas, eGFR estimated glomerular filtration rate (calculated using the Chronic Kidney Disease Epidemiology Collaboration equation), ESKD end-stage kidney disease.

*Subset of study population with non-missing weight at 12 months (or, if unavailable, at 6 months). Weight loss was median 3.5% (IQR 0.6, 6.9%) following GLP1-RA initiation and 0.1% (IQR -2.4, 3.1%) following DPP4i/SU initiation.

†Interaction p=0.59.

‡Double-robust overlap-weighted HR.

Supplemental Table 5. Baseline characteristics at initiation of SU, DPP4i, and GLP1-RA, before and after overlap weighting.

	SGLT2i + SU (n=5,139)	SGLT2i + DPP4i (n=8,481)	SGLT2 + GLP1-RA (20,039)	SMD	SGLT2i + SU (n=5,139)	SGLT2i + DPP4i (n=8,481)	SGLT2 + GLP1-RA (20,039)	SMD
Sociodemographic characteristics								
Age, years (mean, SD)	59 (11)	60 (11)	59 (10)	0.072	60 (12)	59 (12)	60 (10)	0.017
Sex (n, %)				0.030				0.012
Male	3,162 (62%)	5,185 (61%)	11,892 (59%)		3,112 (61%)	5,205 (61%)	12,065 (60%)	
Female	1,977 (38%)	3,296 (39%)	8,147 (41%)		2,027 (39%)	3,276 (39%)	7,974 (40%)	
Ethnicity (n, %)				0.092				<0.01
White	3,870 (75%)	6,468 (76%)	16,192 (81%)		3,991 (78%)	6,543 (77%)	15,590 (78%)	
South Asian	824 (16%)	1,284 (15%)	2,384 (12%)		739 (14%)	1,240 (15%)	2,881 (14%)	
Black	246 (5%)	391 (5%)	761 (4%)		218 (4%)	372 (4%)	824 (4%)	
Other	199 (4%)	338 (4%)	702 (4%)		191 (4%)	326 (4%)	744 (4%)	
Index of multiple deprivation, quintiles (n, %)				0.028				<0.01
1 (least deprived)	786 (15%)	1,396 (16%)	3,218 (16%)		816 (16%)	1,334 (15%)	3,188 (16%)	
2	915 (18%)	1,520 (18%)	3,640 (18%)		917 (18%)	1,532 (18%)	3,645 (18%)	
3	948 (18%)	1,535 (18%)	3,740 (19%)		951 (18%)	1,570 (18%)	3,683 (18%)	
4	1,187 (23%)	1,953 (23%)	4,494 (22%)		1,171 (23%)	1,957 (23%)	4,526 (23%)	

5 (most deprived)	1,303 (25%)	2,077 (24%)	4,948 (25%)		1,284 (25%)	2,088 (25%)	4,997 (25%)	
Laboratory and vital signs measurements								
BMI, kg/m^2 (mean, SD)	32 (7)	32 (7)	35 (7)	0.29	33 (7)	32 (7)	33 (6)	0.055
Systolic blood pressure, mmHg (mean, SD)	130 (13)	130 (13)	130 (13)	0.037	130 (14)	130 (14)	130 (13)	0.013
Total cholesterol, mmol/L (mean, SD)	4.4 (1.2)	4.3 (1.1)	4.3 (1.1)	0.074	4.4 (1.2)	4.4 (1.2)	4.4 (1.2)	<0.01
HbA_{1c}, mmol/mol (mean, SD)	80 (19)	72 (15)	76 (16)	0.32	76 (16)	76 (17)	76 (16)	0.013
HbA_{1c}, % (mean, SD)	9.5 (3.9)	8.7 (3.5)	9.1 (3.6)	0.32	9.1 (3.6)	9.1 (3.7)	9.1 (3.6)	0.013
eGFR, mL/min per 1.73m^2 (mean, SD)	93 (18)	92 (19)	93 (18)	0.045	92 (19)	93 (20)	93 (18)	0.017
eGFR <60 mL/min per 1.73m^2 (n, %)	283 (6%)	611 (7%)	1083 (5%)	0.049	285 (6%)	539 (6%)	980 (5%)	0.015
uACR, mg/mmol (median, IQR)	1.6 [0.8, 3.4]	1.4 [0.8, 3.1]	1.7 [0.9, 3.5]	0.019	1.6 [0.8, 3.4]	1.5 [0.8, 3.2]	1.7 [0.9, 3.3]	0.015
Albuminuria status (n, %)				0.035				<0.01
Normal uACR (<3mg/mmol)	3,766 (73%)	6,312 (74%)	14,462 (72%)		3,779 (74%)	6,267 (74%)	14,733 (74%)	
Low-level uACR (3-30mg/mmol)	1,207 (24%)	1,890 (22%)	4,879 (24%)		1,191 (23%)	1,950 (23%)	4,677 (23%)	
Severely increased uACR (≥30mg/mmol)	166 (3%)	279 (3%)	698 (4%)		169 (3%)	264 (3%)	629 (3%)	

Clinical characteristics								
Diabetes duration at treatment initiation, years (median, IQR)	7.7 [4.6, 11.7]	8.7 [5.1, 13.3]	10.9 [6.9, 15.6]	0.33	8.6 [5.3, 12.8]	8.4 [5.0, 12.7]	8.9 [5.5, 12.9]	0.038
Smoking status (n, %)				0.057				<0.01
Non-smoker	2,608 (51%)	4,375 (52%)	10,047 (50%)		2,627 (51%)	4,309 (51%)	10,211 (51%)	
Ex-smoker	1,664 (32%)	2,836 (33%)	7,086 (35%)		1,712 (33%)	2,833 (33%)	6,678 (33%)	
Current smoker	867 (17%)	1,270 (15%)	2,906 (15%)		800 (16%)	1,339 (16%)	3,150 (16%)	
Atrial fibrillation (n, %)	337 (7%)	591 (7%)	1,321 (7%)	0.011	333 (6%)	551 (6%)	1,304 (7%)	<0.01
Atherosclerotic cardiovascular disease (n, %)	1,164 (23%)	1,956 (23%)	5,085 (25%)	0.043	1,185 (23%)	1,939 (23%)	4,601 (23%)	<0.01
Heart failure (n, %)	342 (7%)	581 (7%)	1,384 (7%)	<0.01	337 (7%)	556 (7%)	1,305 (7%)	<0.01
Electronic frailty index (mean, SD)	0.14 (0.08)	0.15 (0.09)	0.16 (0.09)	0.14	0.15 (0.09)	0.15 (0.09)	0.15 (0.08)	0.019
Frailty category* (n, %)				0.14				0.014
Fit (<0.12)	2,602 (51%)	4,025 (48%)	8,201 (41%)		2,472 (48%)	4,127 (49%)	9,488 (47%)	
Mild frailty (0.12-0.24)	1,881 (37%)	3,192 (38%)	8,347 (42%)		1,948 (38%)	3,168 (37%)	7,765 (39%)	
Moderate frailty (0.24-0.36)	511 (10%)	963 (11%)	2,784 (14%)		564 (11%)	921 (11%)	2,249 (11%)	
Severe frailty (≥0.36)	145 (3%)	301 (4%)	707 (4%)		155 (3%)	265 (3%)	537 (3%)	
Hospitalisation in the year before baseline (n, %)	1,261 (25%)	1,903 (22%)	4,408 (22%)	0.041	1,202 (23%)	1,954 (23%)	4,654 (23%)	<0.01

Medications								
Number of current glucose-lowering treatments (n, %)				0.41				0.010
2	589 (11%)	1,106 (13%)	1,304 (7%)		568 (11%)	918 (11%)	2,200 (11%)	
3	3,690 (72%)	6,044 (71%)	10,359 (52%)		3,568 (69%)	5,978 (70%)	14,050 (70%)	
4+	860 (17%)	1,331 (16%)	8,376 (42%)		1,003 (20%)	1,585 (19%)	3,789 (19%)	
On statin (n, %)	4,551 (89%)	7,507 (89%)	18,323 (91%)	0.065	4,582 (89%)	7,561 (89%)	17,894 (89%)	<0.01
On insulin (n, %)	94 (2%)	416 (5%)	2,522 (13%)	0.29	149 (3%)	258 (3%)	618 (3%)	<0.01
On ACE inhibitor or angiotensin-II receptor blocker (n, %)	3,356 (65%)	5,687 (67%)	14,644 (73%)	0.11	3,439 (67%)	5,676 (67%)	13,522 (67%)	<0.01
Study period								
Calendar year at baseline (n, %)				0.24				<0.01
2013-2016	408 (8%)	796 (9%)	837 (4%)		365 (7%)	620 (7%)	1,487 (7%)	
2017-2018	822 (16%)	1,496 (18%)	2,071 (10%)		796 (15%)	1,283 (15%)	3,117 (16%)	
2019-2020	1,239 (24%)	2,212 (26%)	4,899 (24%)		1,303 (25%)	2,154 (25%)	4,996 (25%)	
2021-2022	2,164 (42%)	3,277 (39%)	10,242 (51%)		2,194 (43%)	3,637 (43%)	8,555 (43%)	
2023	506 (10%)	700 (8%)	1,990 (10%)		481 (9%)	787 (9%)	1,884 (9%)	

Abbreviations: SGLT2i sodium-glucose cotransporter-2 inhibitors, GLP1-RA glucagon-like peptide-1 receptor agonists, DPP4i dipeptidyl peptidase-4 inhibitors, SU sulfonylureas, SMD standardised mean difference, SD standard deviation, IQR interquartile range, BMI body mass index, eGFR estimated glomerular filtration rate (calculated using the Chronic Kidney Disease Epidemiology Collaboration equation), uACR urinary albumin/creatinine ratio.

Weighted characteristics are overlap-weighted values normalised to the original sample size.

*Frailty category based on the electronic frailty index.

Supplemental Table 6. Event rates of the primary, secondary, and safety outcomes per treatment arm (unweighted), as well as unadjusted and double-robust overlap-weighted HRs.

	SGLT2i + GLP1-RA			SGLT2i + DPP4i/SU				
Outcome	Number of individuals at risk	Number of events	Event rate per 1000 person-years	Number of individuals at risk	Number of events	Event rate per 1000 person-years	Unadjusted HR‡ (95%CI)	Double-robust overlap-weighted HR (95%CI)
Kidney disease progression (≥40% eGFR decline, ESKD, or death from kidney-related causes)	20,039	187	6.5	13,620	189	7.8	0.84 (0.69, 1.03)	0.73 (0.58, 0,92)
MACE (incl. CV death)	20,039	860	30.5	13,620	725	30.7	0.97 (0.88, 1.07)	0.89 (0.80, 1.00)
Hospitalisation for HF	20,039	709	25.0	13,620	606	25.4	0.93 (0.83, 1.04)	0.86 (0.76, 0.98)
Acute pancreatitis*	19,727	32	1.1	13,305	25	1.1	1.06 (0.63, 1.79)	0.94 (0.52, 1.70)
Incident diabetic retinopathy†	10,844	1,097	74.9	8,719	936	63.9	1.17 (1.07, 1.27)	1.07 (0.97, 1.18)
Lower limb fracture	20,039	86	3.0	13,620	82	3.4	0.87 (0.64, 1.18)	0.92 (0.65, 1.30)

*In those with no recorded history of prior acute or chronic pancreatitis.

†In those with no recorded history of prior diabetic retinopathy.

‡Unadjusted hazard ratios are presented for transparency but reflect substantial baseline confounding by indication. The double-robust overlap-weighted hazard ratios represent the primary causal estimates, using propensity score weighting combined with covariate adjustment to minimise residual bias.

Abbreviations: SGLT2i sodium-glucose cotransporter-2 inhibitors, GLP1-RA glucagon-like peptide-1 receptor agonists, DPP4i dipeptidyl peptidase-4 inhibitors, SU sulfonylureas, eGFR estimated glomerular filtration rate (calculated using the Chronic Kidney Disease Epidemiology Collaboration equation), ESKD end-stage kidney disease, MACE major adverse cardiovascular events, CV cardiovascular, HF heart failure.

Supplemental Figure 1. Propensity score distribution before and after overlap-weighting. The propensity score represents the probability of GLP1-RA initiation.

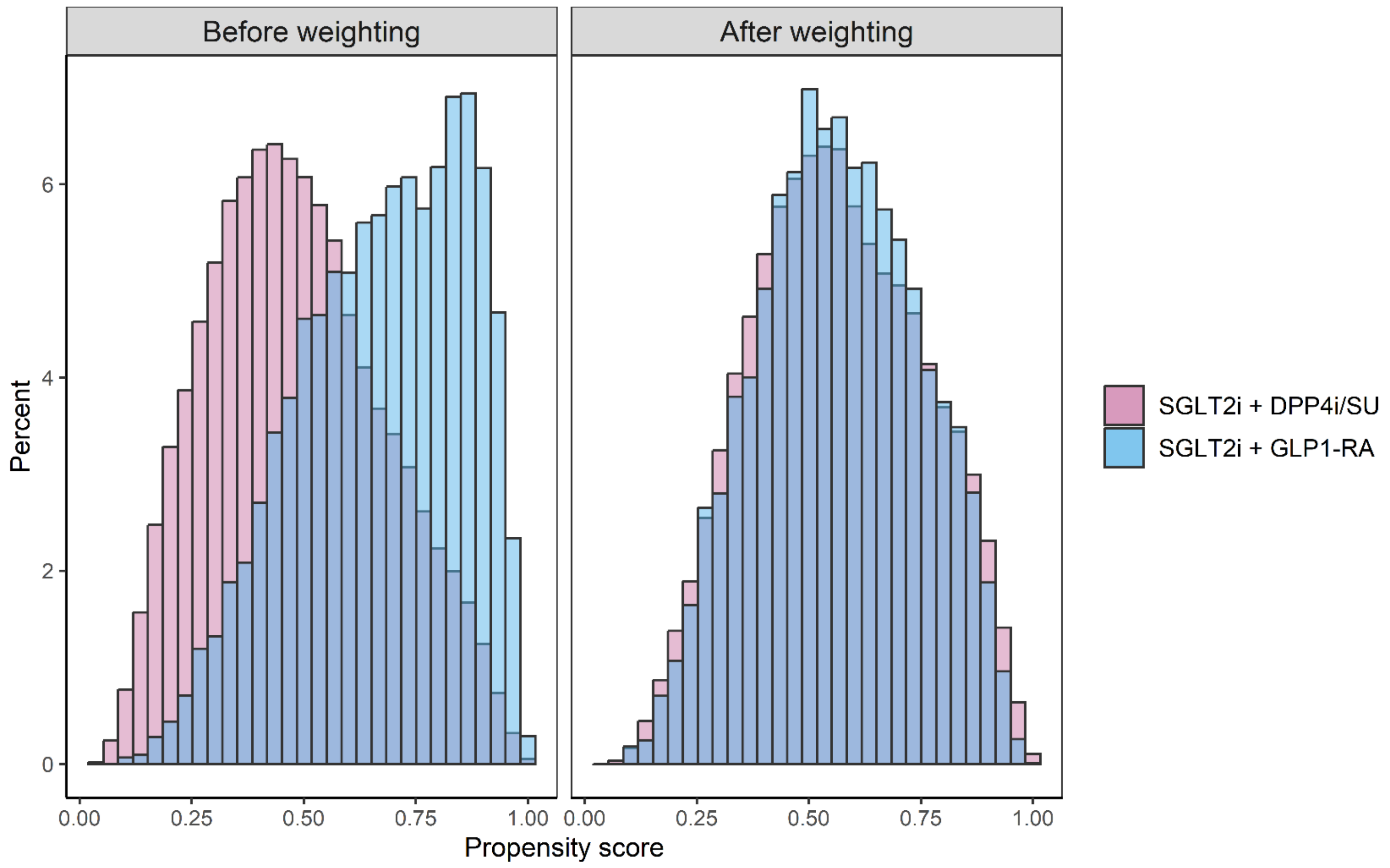

Abbreviations: SGLT2i sodium-glucose cotransporter-2 inhibitors; GLP1-RA glucagon-like peptide-1 receptor agonists, DPP4i DPP4i-inhibitors; SU sulfonylureas**.**

Supplemental Figure 2. Forest plot of the HRs for kidney disease progression (composite of ≥40% eGFR decline, ESKD, or death from kidney-related causes) for GLP1-RA initiation compared with DPP4i/SU initiation across different analysis approaches. The top row shows the primary analysis HR for reference, estimated using double-robust overlap-weighting with a maximum follow-up of 3 years under an intention-to-treat framework.

Kidney disease progression	SGLT2i + GLP1-RA (n/N)	SGLT2i + DPP4i/SU (n/N)	HR* (95% CI)
Primary analysis	187 / 20,039	189 / 13,620	0.73 (0.58, 0.92)
Follow-up extended up to 5 years	239 / 20,039	255 / 13,620	0.72 (0.59, 0.88)
Per-protocol analysis	143 / 20,039	146 / 13,620	0.69 (0.53, 0.89)
Multivariable adjustment only	187 / 20,039	189 / 13,620	0.72 (0.58, 0.91)
Inverse probability of treatment weighting	187 / 20,039	189 / 13,620	0.77 (0.61, 0.97)
Fine-Gray competing risk analysis (overlap-weighted)	187 / 20,039	189 / 13,620	0.74 (0.59, 0.93)

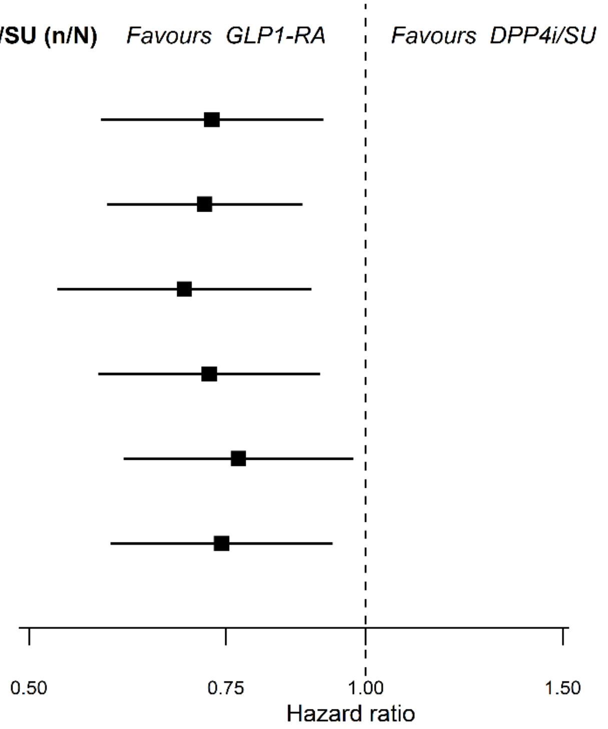

Abbreviations: eGFR estimated glomerular filtration rate, ESKD end-stage kidney disease, SGLT2i sodium-glucose cotransporter-2 inhibitors; GLP1-RA glucagon-like peptide-1 receptor agonists, DPP4i DPP4i-inhibitors; SU sulfonylureas, n/N number of events/number of subjects.

*Double-robust overlap-weighted HRs (except where specified as multivariable adjusted alone or inverse probability of treatment weighted).

Supplemental Figure 3. Forest plot of the overlap-weighted HRs for kidney disease progression (composite of ≥40% eGFR decline, ESKD, or death from kidney-related causes) for GLP1-RA initiation compared with DPP4i/SU initiation when excluding and including selected patient subgroups. The top row shows the primary study sample HR for reference.

Kidney disease progression	SGLT2i + GLP1-RA (n/N)	SGLT2i + DPP4i/SU (n/N)	HR* (95% CI)
Primary study sample	187 / 20,039	189 / 13,620	0.73 (0.58, 0.92)
Excluding GLP1-RA initiation if previous DPP4/SU initiation	175 / 18,030	189 / 13,620	0.75 (0.60, 0.95)
Excluding individuals treated with insulin	139 / 17,517	172 / 13,110	0.69 (0.54, 0.89)
Excluding individuals treated with >4 glucose-lowering treatments	171 / 19,061	187 / 13,553	0.73 (0.58, 0.92)
Above exclusions combined	123 / 15,134	171 / 13,080	0.72 (0.56, 0.94)
Excluding individuals without linked hospital inpatient data	135 / 16,304	149 / 11,097	0.66 (0.51, 0.86)
Including individuals with missing baseline uACR	257 / 24,601	258 / 17,197	0.76 (0.62, 0.92)

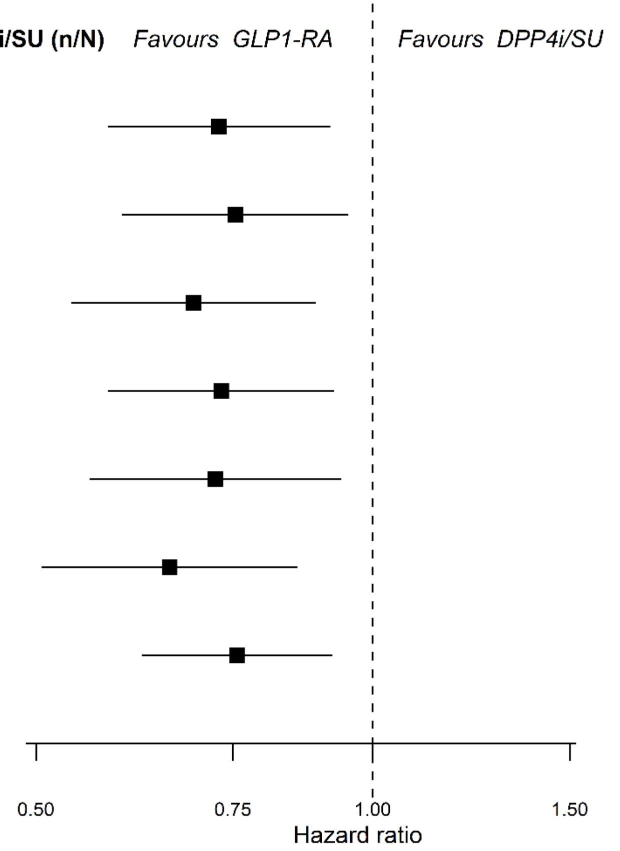

Abbreviations: eGFR estimated glomerular filtration rate, ESKD end-stage kidney disease, SGLT2i sodium-glucose cotransporter-2 inhibitors; GLP1-RA glucagon-like peptide-1 receptor agonists, DPP4i DPP4i-inhibitors; SU sulfonylureas, n/N number of events/number of subjects, uACR urine albumin/creatinine ratio.

*Double-robust overlap-weighted HRs.

Supplemental Figure 4. Forest plot of the overlap-weighted HRs of the primary outcome and secondary outcomes for GLP1-RA initiation compared with DPP4i initiation (Panel A), for GLP1-RA initiation compared with SU initiation (Panel B), and for DPP4i initiation compared with SU initiation (Panel C).

A.

**Outcome**	**SGLT2i + GLP1-RA (n/N)**	**SGLT2i + DPP4i (n/N)**	**HR* (95% CI)**
Kidney disease progression	187 / 20,039	126 / 8,481	0.70 (0.52, 0.93)
MACE (incl. CV death)	860 / 20,039	457 / 8,481	0.90 (0.78, 1.03)
Hospitalisation for HF	709 / 20,039	374 / 8,481	0.89 (0.76, 1.04)
Acute pancreatitis	32 / 19,727	16 / 8,308	0.90 (0.46, 1.74)
Incident diabetic retinopathy	1,097 / 10,844	562 / 5,264	1.05 (0.94, 1.18)
Lower limb fracture	86 / 20,039	52 / 8,481	0.87 (0.58, 1.32)

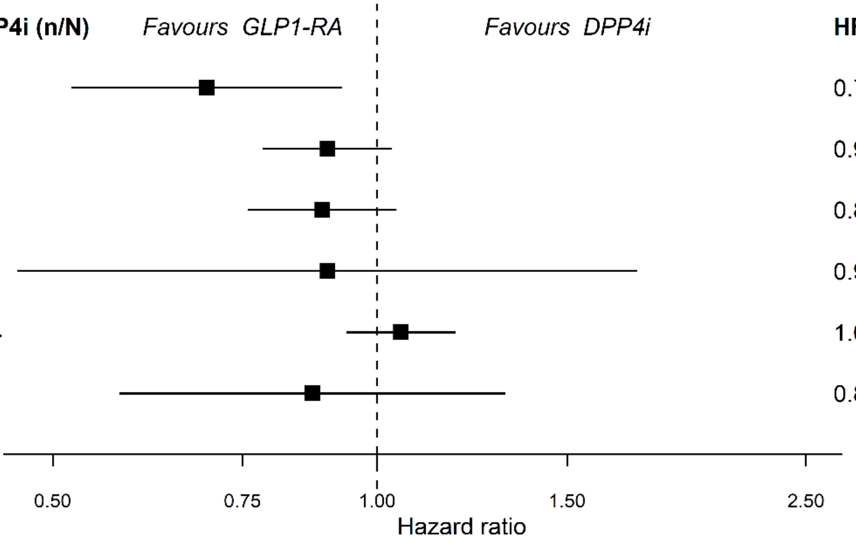

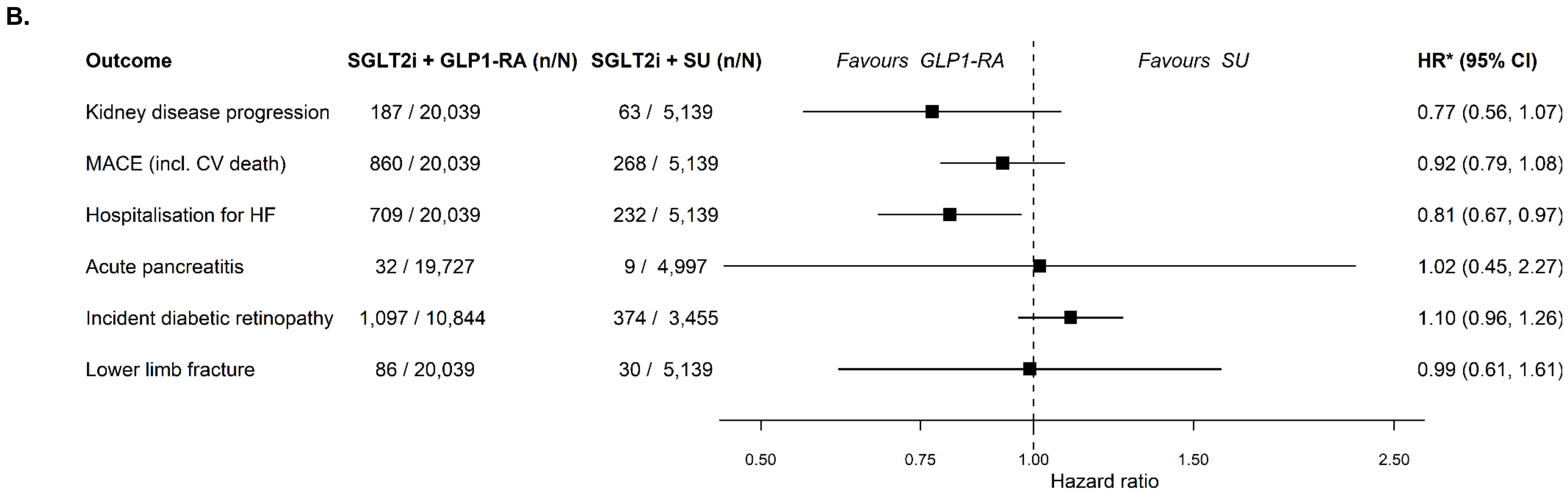
B.
Outcome
SGLT2i + GLP1-RA (n/N)
SGLT2i + SU (n/N)
Favours GLP1-RA
Favours SU
HR* (95% CI)
Kidney disease progression
187 / 20,039
63 / 5,139
0.77 (0.56, 1.07)
MACE (incl. CV death)
860 / 20,039
268 / 5,139
0.92 (0.79, 1.08)
Hospitalisation for HF
709 / 20,039
232 / 5,139
0.81 (0.67, 0.97)
Acute pancreatitis
32 / 19,727
9 / 4,997
1.02 (0.45, 2.27)
Incident diabetic retinopathy
1,097 / 10,844
374 / 3,455
1.10 (0.96, 1.26)
Lower limb fracture
86 / 20,039
30 / 5,139
0.99 (0.61, 1.61)
0.50
0.75
1.00
1.50
2.50
Hazard ratio

C.

Outcome	SGLT2i + DPP4i (n/N)	SGLT2i + SU (n/N)	HR* (95% CI)
Kidney disease progression	126 / 8,481	63 / 5,139	1.12 (0.81, 1.53)
MACE (incl. CV death)	457 / 8,481	268 / 5,139	1.03 (0.87, 1.21)
Hospitalisation for HF	374 / 8,481	232 / 5,139	0.91 (0.75, 1.09)
Acute pancreatitis	16 / 8,308	9 / 4,997	1.13 (0.49, 2.59)
Incident diabetic retinopathy	562 / 5,264	374 / 3,455	1.04 (0.91, 1.20)
Lower limb fracture	52 / 8,481	30 / 5,139	1.14 (0.69, 1.86)

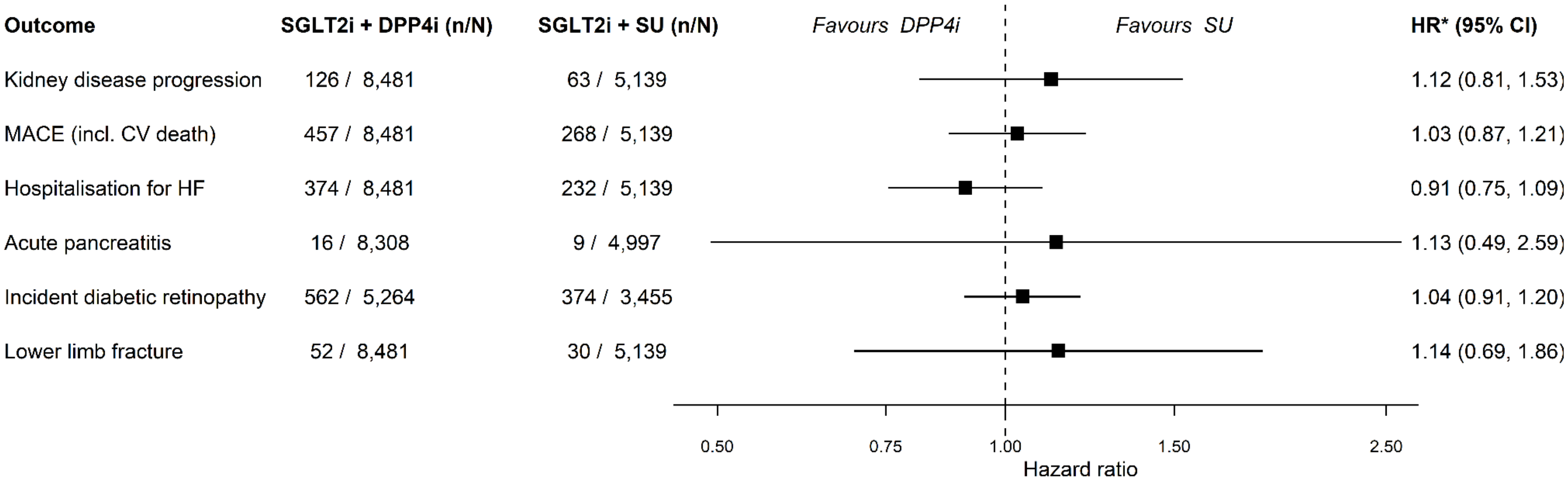

Abbreviations: eGFR estimated glomerular filtration rate, ESKD end-stage kidney disease, SGLT2i sodium-glucose cotransporter-2 inhibitors; GLP1-RA glucagon-like peptide-1 receptor agonists, DPP4i DPP4i-inhibitors; SU sulfonylureas, n/N number of events/number of subjects, MACE major adverse cardiovascular events, CV cardiovascular, HF heart failure.

*Double-robust overlap-weighted HRs.
